# The Effect of Warfarin Use on Postoperative Outcomes after Femoral Neck Surgery

**DOI:** 10.3390/jcm12041307

**Published:** 2023-02-07

**Authors:** Jeremy Dubin, Esequiel Palmanovich, Eitan Iohanes, Ronen Blecher, David Segal, Yaron Brin, Michael Drexler, Ran Atzmon

**Affiliations:** 1 Tel Aviv Medical Center, Department of Orthopaedic Surgery, Affiliated with the Sackler Faculty of Medicine and Tel Aviv University, Tel Aviv 6423906, Israel; 2 Orthopaedic Department, Meir Medical Center, Kfar Saba, Affiliated to Sackler Faculty of Medicine, Tel-Aviv University, Tel Aviv 6423906, Israel; 3 Assuta Medical Center, Department of Orthopaedic Surgery, Affiliated with the Faculty of Health and Science and Ben Gurion University, Ashdod 7747629, Israel

**Keywords:** warfarin, mortality rate, femoral neck, anticoagulation, hospitalization days

## Abstract

**Introduction**: Anticoagulation use in the elderly is common for patients undergoing femoral neck hip surgery. However, its use presents a challenge to balance it with associated comorbidities and benefits for the patients. As such, we attempted to compare the risk factors, perioperative outcomes, and postoperative outcomes of patients who used warfarin preoperatively and patients who used therapeutic enoxaparin. **Methods**: From 2003 through 2014, we queried our database to determine the cohorts of patients who used warfarin preoperatively and the patients who used therapeutic enoxaparin. Risk factors included age, gender, Body Mass Index (BMI) > 30, Atrial Fibrillation (AF), Chronic Heart Failure (CHF), and Chronic Renal Failure (CRF). Postoperative outcomes were also collected at each of the patients’ follow-up visits, including number of hospitalization days, delays to theatre, and mortality rate. **Results**: The minimum follow-up was 24 months and the average follow-up was 39 months (range: 24–60 months). In the warfarin cohort, there were 140 patients and 2055 patients in the therapeutic enoxaparin cohort. Number of hospitalization days (8.7 vs. 9.8, *p* = 0.02), mortality rate (58.7% vs. 71.4%, *p* = 0.003), and delays to theatre (1.70 vs. 2.86, *p* < 0.0001) were significantly longer for the anticoagulant cohort than the therapeutic enoxaparin cohort. Warfarin use best predicted number of hospitalization days (*p* = 0.00) and delays to theatre (*p* = 0.01), while CHF was the best predictor of mortality rate (*p* = 0.00). Postoperative complications, such as Pulmonary Embolism (PE) (*p* = 0.90), Deep Vein Thrombosis (DVT) (*p* = 0.31), and Cerebrovascular Accidents (CVA) (*p* = 0.72), pain levels (*p* = 0.95), full weight-bearing status (*p* = 0.08), and rehabilitation use (*p* = 0.34) were similar between the cohorts. **Conclusion:** Warfarin use is associated with increased number of hospitalization days and delays to theatre, but does not affect the postoperative outcome, including DVT, CVA, and pain levels compared to therapeutic enoxaparin use. Warfarin use proved to be the best predictor of hospitalization days and delays to theatre while CHF predicted mortality rate.

## 1. Introduction

Each year, at least 300,000 adults ages 65 and older are hospitalized for hip fractures [1]. More than 90% of hip fracture patients are older than 65 years and usually present with comorbidities, such as diabetes mellitus, chronic pulmonary disease, dementia, cancer, and thyroid disease [2,3,4]. Anticoagulants are often used for their effectiveness in reducing the risk of ischemic stroke, pulmonary embolism, and deep vein thrombosis, but can lead to increased risk of hemorrhages [5]. The main risk factors for anticoagulant use in hip fracture include cardiac arrhythmia, previous stroke, and thromboembolic event.

Anticoagulation use, such as warfarin (also known by its trade name as Coumadin), is used in a large number of high-risk patients, including for patients with prosthetic valves, antiphospholipid syndrome, or a high risk for gastrointestinal bleeding [6]. Warfarin can prevent arterial and venous thrombosis by inhibiting vitamin K epoxide reductase, which reduces the synthesis of active clotting factors. In addition, warfarin can be effectively reversed using vitamin K to correct coagulopathy to provide flexibility for its use by the medical staff [7]. The management of warfarin in hip fractures remains multifaceted and depends on the interactions between relevant risk factors for anticoagulant, as among them are atrial fibrillation and cardiovascular disease; perioperative events, such as delays to surgery and surgical fixation; and postoperative outcomes, such as hospitalization duration and mortality rate.

Research has shown that warfarin use prior to admission for hip fracture may lead to delays in surgery, increased length of stay in the hospital, and higher mortality rates compared to patients who did not use anticoagulants [8,9]. However, other studies found that anticoagulation with warfarin did not significantly affect time to surgery or length of stay compared to non-warfarin anticoagulation [10,11]. Delays in surgery can result in additional costs of USD 2638 per patient and potentially worse patient outcomes, such as pressure ulcers [12,13].

This study aimed to better understand the differences in warfarin risk factors, perioperative events, and postoperative events between patients on therapeutic warfarin anticoagulation, compared to patients who took therapeutic enoxaparin. Specifically, we examined (i) potential risk factors between the cohorts, including past medical history and type of surgical fixation, (ii) interactions between risk factors using a multivariate regression analysis, and (iii) postoperative outcomes, including number of hospitalization days, delays to theatre, and morality rate.

## 2. Methods

**Figure 1:**
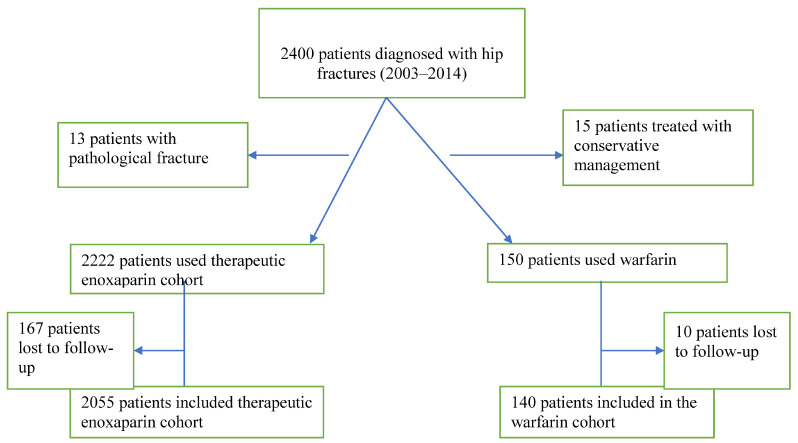
Inclusion criteria for patient selection.

We performed a retrospective review of prospectively collected data from 2003–2014 in a single center to determine the patients who received therapeutic warfarin anticoagulation, and the patients who received therapeutic enoxaparin before surgery. The inclusion criteria included: (1) diagnosis of femoral neck fracture via surgical route and (2) minimum follow-up of 24 months. The exclusion criteria included: (1) non-operative hip fracture; (2) contraindications for anticoagulation use, including bleeding abnormalities, malignant hypertension, or advanced retinopathy; and (3) patients who were diagnosed with pathological fractures (Figure 1). The study was approved by the local institutional review board as part of several different studies done on the same population [14].

### 2.1. Anticoagulation Protocol

Our institution provides oral prophylaxis based on surgical preference. The normal protocol includes oral enoxaparin in 40 mg subcutaneous once/day, including for atrial fibrillation. Therapeutic enoxaparin is set at 1.5 mg/kg/day. Warfarin is utilized for patients with prosthetic valves, antiphospholipid syndrome, or a high risk for gastrointestinal bleeding, such as history of peptic ulcer disease, chronic renal failure, diabetes mellitus, concomitant use of other platelet agents, steroids, and nonsteroidal anti-inflammatory drugs [15]. Patients were given either 3 g/day or 5 g/day of warfarin depending on individual weight leading up to the surgery. When warfarin and enoxaparin are used together postoperatively, enoxaparin is stopped and oral warfarin is continued alone on postoperative day 2 or 3 until target INR is reached. An anticoagulant service allows for daily monitoring of all patients using a warfarin-initiation guideline, which targets an INR range of 2–3. Patients stopped their regiment 5–6 days before surgery, and continued taking it on the following day 12 h after surgery. Bridging anticoagulation, which included enoxaparin, was started 3 days before surgery, and stopped 24 h before surgery. All patients taking warfarin with an International Normalized Ratio (INR) > 1.5 were actively reversed to <1.5 using vitamin K. This process can take up to four days for the INR to reach adequate levels. Postoperatively, vitamin K, in a dose of 2.5 to 10 mg, was given if INR > 5 or hip fractures that are not treated within 48 h, as per national guidelines. Warfarin is continued for 21 days or until hospital discharge. There have been no reported cases of overdose, preoperative bleeding due to warfarin, or bleeding episodes.

### 2.2. Risk Factors

At the patients’ preoperative visit, the physician collected data on risk factors that may mediate anticoagulation use. The risk factors included age, gender, Body Mass Index (BMI) > 30, Atrial Fibrillation (AF), Chronic Heart Failure (CHF), Chronic Renal Failure (CRF), Hypertension (HTN), Ischemic Heart Disease (IHD), Hypocholesterolemia (HCL), Diabetes Mellitus (DM), Pulmonary Embolism (PE), Deep Vein Thrombosis (DVT), Cerebrovascular Accident (CVA), type of surgery, and type of fixation.

### 2.3. Postoperative Outcomes

Outcomes were collected by the physician at the patients’ respective follow-up visit (6 weeks, 3 months, 6 months, 1 year, and each subsequent year). The minimum follow-up was 24 months and the average follow-up was 39 months (range: 24–60 months). The postoperative outcomes included number of hospitalization days, days from admission to surgery, occurrence of PE, DVT, CVA, pain levels, weight-bearing status, discharge to designated rehabilitation center versus solely physiotherapy, and mortality rate.

### 2.4. Statistical Analysis

Statistical analysis was performed by applying IBM SPSS 21 (IBM, Armonk, New York, NY, USA, 2016). A *p*-value of <0.05 with 95% confidence interval was viewed as statistically significant. All variables with a *p*-value < 0.05 from the univariate analysis were used in the multivariate logistic regression model. Normally distributed continuous data were compared using Student’s *t*-test.

## 3. Results

There were 150 patients in the warfarin cohort and 2222 patients in the therapeutic enoxaparin cohort. In the warfarin cohort, 7 patients (4.7%) were lost to follow-up and 10 patients were lost to follow-up (2.2%) in the therapeutic enoxaparin cohort, leading to 140 patients in the warfarin cohort and 2055 patients in the therapeutic enoxaparin. The minimum follow-up was 24 months with an average follow-up of 39 months (range: 24–60 months). We found no significant differences in terms of age (*p* = 0.20), gender breakdown (*p* = 0.09), and BMI > 30 (*p* = 0.12). In the therapeutic enoxaparin cohort, the average age was 80.61 years with 666 males and 1389 females, with 3.8% of the patients having a BMI greater than 30. In the warfarin cohort, the average age was 79 years with 55 males, 85 females, and with 6.4% of the patients having BMI greater than 30

We found several statistically significant risk factors for warfarin use between the cohorts in both the univariate analysis and the multivariate analysis. AF (10% vs. 80.7%, *p* < 0.0001 for univariate, and *p* = 0.00 for multivariate), CHF (10.4% vs. 35.7%, *p* < 0.0001 for univariate, and *p* = 0.004 for multivariate), HCL (25.3% vs. 37.9%, *p* = 0.001 for univariate, and *p* = 0.004 for multivariate), and DVT (0.2% vs. 5.7%, *p* < 0.0001, *p* = 0.00 for multivariate) were significantly higher in the warfarin cohort. CRF (9.9% vs. 20.7%, *p* = 0.005 for univariate, and *p* = 0.31 for multivariate) and IHD (26.5% vs. 43.6%, and *p* < 0.0001 for univariate, *p* = 0.35) only showed percentages in the univariate analysis and not the multivariate analysis (Table 1).

The type of surgical diagnosis (*p* = 0.71) and type of fixation (*p* = 0.50) were not statistically significant in the univariate analysis. We found no significant difference between types of surgical diagnosis, regarding anticoagulation use (Table 2). Additionally, there was no significant difference in terms of individual surgical fixations between the cohorts (Table 3).

In comparing postoperative outcomes between cohorts, there were statistically significant differences in the univariate and multivariate analysis, including hospitalization days, mortality rate, and delays to theatre. We found that number of hospitalization days (8.7 vs. 9.8, *p* = 0.02), mortality rate (58.7% vs. 71.4%, *p* = 0.003), and delays to theatre (1.70 vs. 2.86, *p* < 0.0001) were significantly longer for the warfarin cohort than the therapeutic enoxaparin cohort. Postoperative complications, such as PE (*p* = 0.90), DVT (*p* = 0.31), and CVA (*p* = 0.72), pain levels (*p* = 0.95), full weight-bearing status (*p* = 0.08), and rehabilitation use (*p* = 0.34) were not found to be significantly different between the cohorts (Table 4).

Multiple linear regression of the statistically significant postoperative outcomes showed that warfarin use best predicted number of hospitalization days (*p* = 0.00) and delays to theatre (*p* = 0.01), while CHF was the best predictor of mortality rate (*p* = 0.00) (Table 5).

## 4. Discussion

The elderly population is suffering from proximal femoral fractures at a high rate. At the same time, the use of warfarin in stroke prevention in the elderly population is steadily increasing as well. These patients often present with comorbidities or preexisting medical problems that can lead to a higher risk for complications, increased length of stay, and increased mortality [16,17,18,19,20,21]. To our knowledge, this is the first study to compare postoperative outcomes after proximal femoral fracture in comparing warfarin use to therapeutic enoxaparin use. The literature supports the use of enoxaparin over warfarin in minimizing postoperative complications, such as DVT and PE [22,23,24,25,26]. Our principal finding was that patients in the warfarin cohort spent more days in the hospital on average and had more delays-to-theatre rate while both groups had similar postoperative functional levels, pain levels, rehabilitation use, and postoperative outcomes, including PE, DVT, and CVA at an average of 39 months’ follow-up.

The impact of warfarin use on hospital stays has been ambiguous in the literature. In our analysis, warfarin use significantly increased the number of hospitalization days compared to therapeutic enoxaparin use (9.8 days vs. 8.7 days, *p* = 0.02). Lawrence et al. [17] found warfarin users were hospitalized 2.0 days longer than patients who were not using anticoagulants. While they controlled for American Society of Anesthesiologists (ASA) score and admission Abbreviated Mental Test score (AMTS), we controlled for BMI (Table 1) and past medical history (Table 1). Nonetheless, the results were similar. Additionally, Taranu et al. [27] and Ranhoff et al. [28] both showed that patients using anticoagulants stay longer in the hospital than nonanticoagulated patients of the same age and injury. However, Eardley et al. [10] and Hoerlyck et al. [9] showed no association between length of stay and use of warfarin for hip fracture in a study of 1024 patients and 2307 patients, respectively [9,10]. Both studies did not control for surgery type, fixation type, and BMI that could play a role in the respective relationship. We maintain the novelty in the choice of including risk factors in the multivariate analysis in showing the major factor that led to increased hospital stay was warfarin use in our study (Table 5). Based on this finding, active steps, such as perioperative warfarin protocols to better manage the timing of warfarin dosing, should be implemented to potentially overcome the increased length of hospital stay [29].

In addition to hospital stay, delays to theatre remains an important variable for consideration because of its association with increased costs and adverse postoperative complications, including deep vein thrombosis, pneumonia, and urinary tract infections [30]. Ranhoff et al. [28] reported that there was longer waiting time for surgery for warfarin users compared with nonusers (23 vs. 12 h, *p* < 0.001). Lawrence et al. [17] found patients taking warfarin were significantly less likely to go to surgery by 36 h and 48 h compared to patients not taking warfarin. This relationship may be attributed to the potential for reversal of anticoagulation to prevent excessive bleeding at the time of surgery [31]. It may take up to four days for INR to reach acceptable levels as was noted in our anticoagulation protocol. In our analysis, warfarin use was the most significant factor that affected delays to theatre, when accounting for demographics, risk factors, and surgical methods. It would be beneficial to subdivide delays to theatre into more specific time points to address the true cause of delays and efforts to address it, including early warfarin reversal and better treatment of complex patients [16].

We found no difference between the warfarin and therapeutic enoxaparin groups regarding perioperative events, such as surgical fixation and fraction, and postoperative events, such as PE, DVT, CVA, pain levels, and rehabilitation use. This finding adds to the varied results of anticoagulant use after hip fractures. Horelyck et al. [9] similarly found no difference between warfarin cohort and non-warfarin cohort regarding postoperative outcomes, including stroke, myocardial infarction, and renal failure, but found there was an increase in renal failure (14% vs. 19%, *p* = 0.04) and heart failure (1% vs. 5%, *p* < 0.01) in the anticoagulation group. A meta-analysis by Xu et al. [31] including twenty-one studies involving 21,417 patients undergoing hip fractures showed that the rates of postoperative thromboembolism were similar regardless of anticoagulation status at the expense of increased blood loss. Lott et al. [32] found that anticoagulation status does not independently increase the risk of surgical outcomes and cost of hospitalization based on their finding that after similar mean number of complications and in-patient mortality rates between the two groups. Lott et al. [32] attributed the outcomes to the comorbidities of the patient on anticoagulation as measured by the Charlson comorbidity index (CCI). Based on our findings and review of the literature, it seems the anticoagulant use has a role in limiting the negative consequences of postoperative outcomes.

Several factors can influence the mortality rate after hip fractures, including delayed surgical treatment and preadmission comorbidities [33,34]. Caruso et al. found that warfarin therapy for proximal femoral fractures is associated with 42% higher risk of death within the first year from surgery [18]. Lawrence et al. [17] also showed a higher mortality for patients taking warfarin (66% vs. 76%, *p* < 0.001) at 12 months. We found a higher mortality rate at 39 months for (58.7% vs. 71.4%, *p* = 0.003) for patients taking warfarin compared to therapeutic enoxaparin use, which may be attributed in part to surgical delays (Table 4). However, in our study, CHF proved to be a bigger risk factor of mortality rate than warfarin use (Table 5). The relationship between CHF and mortality rate in hip fractures and its comparative value to other preoperative risk factors in the analysis remains unclear [35] but should be given serious consideration given the findings. Since we did not find a difference in postoperative outcomes between the cohorts, we believe the mortality rate was either a result of perioperative events or preoperative risk factors (CHF). The effect of perioperative outcomes may be mitigated due to the uniform treatment of warfarin use, which was supported by finding no difference in the type of diagnosis or type of surgical fixation between the cohorts.

## 5. Limitation

We acknowledge several limitations in the study. We were not inclusive of all risk factors that could play a role in contributing to postoperative outcomes in the warfarin cohort. The ASA grade and preoperative mental assessments was not recorded. However, the similarities in the prevalence of risk factors that were addressed help to reduce this concern. Though we performed a retrospective analysis, the multivariate analysis and the relatively high number of patients may strengthen the results by providing better indications of the influence of anticoagulant use in this study. Theoretically, the variable treatment regarding postoperative treatment between the groups could explain the difference in length of stay, but we found no difference in rehabilitation as well as postoperative outcomes, which supports the effect of risk factors, such as CHF, on mediating the relationship of warfarin and mortality rate. While we found that postoperative outcomes were similar among the groups despite an increased length of stay and delays to theatre, we were not able to assess this relationship to a larger extent. The similar outcomes may be attributed to the hospital’s management of anticoagulation [10], specifically the reversal of warfarin with an INR > 1.5. This should be examined in more detail in a follow-up study to understand the impact of warfarin use on postoperative outcomes. A long-term follow-up would better substantiate the relationship between risk factors and mortality rate, but a novel finding at 39 months’ follow-up is a step toward reaching that time point. In a future study, including the timing of warfarin use and subdivision of delays to theatre would be useful in improving its role in the health of patients undergoing femoral neck surgery. An area to examine in the future may include the relationship between acute respiratory syndrome (SARS) caused by coronavirus (SARS-CoV) and pulmonary embolism, femur fractures, and anesthesia [36,37]. Additionally, exploring the impact of warfarin use directly on postoperative outcomes since this study provides an impetus for the observation and may even be a potential benefit of warfarin use.

## 6. Conclusions

Warfarin use may lead to increased number of hospitalization days and delays to theatre when compared with therapeutic enoxaparin use in femoral neck surgery. It becomes a goal of the physician and hospital team to better manage warfarin use in these patients. This includes understanding that risk factors play a role that is equally important as anticoagulant use itself in affecting the mortality rate.

## Figures and Tables

**Table 1:** Risk factors for anticoagulant use between cohorts.

	Therapeutic Enoxaparin Use	Warfarin Use	*p*-Value	Adjusted *p*-Value
Atrial fibrillation	206 (10.0%)	113 (80.7%)	*p* < 0.0001	0.00
Chronic heart failure	214 (10.4%)	50 (35.7%)	*p* < 0.0001	0.004
Chronic renal failure	203 (9.9%)	29 (20.7%)	0.0005	0.31
Hypertension	1301 (63.3%)	100 (71.4%)	0.05	N/A
Ischemic heart disease	545 (26.5%)	61 (43.6%)	*p* < 0.0001	0.35
Hypocholesteremia	520 (25.3%)	53 (37.9%)	0.001	0.004
Diabetes mellitus	561 (27.2%)	31 (22.1%)	0.14	N/A
Pulmonary embolism	20 (0.9%)	4 (2.9%)	0.77	N/A
Deep vein thrombosis	5 (0.2%)	8 (5.7%)	*p* < 0.0001	0.00
Cerebrovascular accident	257 (12.5%)	19 (13.6%)	0.74	N/A
Type of surgery	N/A	N/A	0.71	N/A
Type of fixation	N/A	N/A	0.50	N/A

**Table 2:** Type of diagnosis between cohorts.

	Therapeutic Enoxaparin Use	Warfarin Use	*p*-Value
Subcapital	812 (39.5%)	51 (36.4%)	0.47
Pertrochanteric	888 (43.2%)	65 (46.4%)	0.46
Basicervical	153 (7.4%)	8 (5.7%)	0.44
Midcervical	22 (1.1%)	1 (0.7%)	0.69
Subtrochanteric	167 (8.1%)	14 (10.0%)	0.44

**Table 3:** Type of surgical fixation and diagnosis between cohorts.

	Therapeutic Enoxaparin Use	Warfarin Use	*p*-Value
Thompson HA	492 (23.9%)	29 (20.7%)	0.38
Bipolar HA	69 (33.6%)	5 (3.6%)	0.89
Richard’s nailing	553 (26.9%)	38 (27.1%)	0.95
Proximal femoral nail	405 (19.7%)	29 (20.7%)	0.77
Proximal femoral nail long	33 (1.6%)	3 (2.1%)	0.62
Percutaneous compression plate	76 (3.7%)	6 (4.3%)	0.72
Dynamic hip screw	115 (5.6%)	10 (7.1%)	0.44
Intramedullary nail fixation	124 (6.0%)	9 (6.4%)	0.84
Cannulated screw	167 (8.1%)	9 (6.4%)	0.47

**Table 4:** Postoperative outcomes between cohorts.

	Therapeutic Enoxaparin Use	Warfarin Use	*p*-Value
Number of hospitalization days, average	8.7	9.8	0.02
Delays to theatre	1.70	2.86	*p* < 0.0001
Pulmonary embolism	27 (1.3%)	2 (1.4%)	0.90
Deep vein thrombosis	46 (2.2%)	5 (3.6%)	0.31
Cerebrovascular accident	102 (5.0%)	6 (4.3%)	0.72
Pain levels	0.90	0.93	0.95
Full weight-bearing status	1755 (85.4%)	112 (80%)	0.08
Rehabilitation use	682 (33.2%)	52 (37.1%)	0.34
Mortality rate	1207 (58.7%)	100 (71.4%)	0.003

**Table 5:** Greatest predictor of postoperative outcomes.

	Greatest Risk Factor	*p*-Value
Number of days of hospitalization	Warfarin use	0.00
Operative delay	Warfarin use	0.01

Controlled for significant preoperative outcomes in the multivariate analysis (AF, CHF, Hypocholesteremia, DVT).
